# Long-Segment Epidural Hemorrhage of the Cervical and Dorsal Spine: A Case Report of a Rare Complication of Dengue Virus Disease

**DOI:** 10.7759/cureus.33435

**Published:** 2023-01-06

**Authors:** Chandrashekar Patil, Akhila Bandaru, Rohit Bandari, Mahesh Kumar, Prashanth Kumar

**Affiliations:** 1 Radiodiagnosis, Malla Reddy Medical College for Women, Hyderabad, IND; 2 Neurology, Malla Reddy Narayana Multispeciality Hospital, Hyderabad, IND; 3 Neurological Surgery, Malla Reddy Narayana Multispeciality Hospital, Hyderabad, IND

**Keywords:** quadriparesis, gradient echo, dengue, epidural hemorrhage, magnetic resonance imaging ( mri)

## Abstract

Dengue hemorrhagic fever is a severe form of dengue virus disease, characterized by minor to major bleeding, thrombocytopenia, and plasma leakage. Common hemorrhagic manifestations include epistaxis, gum bleeding, gastrointestinal bleeding, hypermenorrhea, and hematuria. Intracranial hemorrhage is one of the most fatal manifestations of central nervous system involvement by dengue disease which is a part of the expanded dengue syndrome. Here we present a case of A 37-year-old male patient who presented with complaints of intermittent high-grade fever and generalized weakness four days prior to consultation. Laboratory investigations revealed mild thrombocytopenia and positive dengue serology. Magnetic resonance imaging of the brain and spine revealed mild diffuse subarachnoid hemorrhage in bilateral parieto-occipital lobes with long segment cervical and dorsal spinal epidural hemorrhage.

## Introduction

Dengue fever is a mosquito-borne viral disease transmitted to humans by *Aedes* mosquitoes, mainly *Aedes aegypti*. It is caused by four viral serotypes (DENV1 to DENV4) which belong to the genus *Flavivirus* and family *Flaviviridae*. It is more common in tropical and subtropical regions. Over the past three decades, dengue has become more common worldwide. Dengue infection can range from subclinical to symptomatic with clinical dengue diseases such as dengue fever, dengue hemorrhagic fever (DHF), dengue encephalitis, and expanded dengue syndrome (EDS). Dengue hemorrhagic fever is a more severe form of dengue virus disease. Plasma leakage and intrinsic coagulopathy are the main pathophysiological changes involved in DHF. Expanded dengue syndrome is a phenomenon that includes the unusual manifestations of dengue causing severe damage to the liver, kidneys, bone marrow, heart, and brain [1]. Cases of EDS have been documented more frequently recently. One of the central nervous system involvement of dengue in EDS is intracranial hemorrhage [2]. Development of active bleeding with moderate thrombocytopenia and normal clotting profile may be explained by platelet functional defects which are known to occur during dengue infection [3].

## Case presentation

A 37-year-old male presented with on-and-off fever (102 degrees F) for four days and generalized weakness in both upper and lower limbs. All necessary investigations were performed. On evaluation, the patient was diagnosed with dengue fever with thrombocytopenia. On examination, the patient was conscious and coherent with the following vital signs: blood pressure of 180/100 mmHg, pulse rate of 54 bpm, and saturation of peripheral oxygen (Spo2) of 95%. Neurological examination revealed sensory loss below the T4 level with absent deep tendon reflexes. The patient had a Glasgow coma score (GCS) of 15 without any signs of meningeal irritation. The pupillary size and reaction were normal. Serological tests for malaria and typhoid infection were negative. He had a hematocrit of 46%, and thrombocytopenia (1.01 lakhs/cumm) with an increased total leukocyte count (19,400 cells/cumm) and a normal coagulation profile (prothrombin time (PT) 14.1 sec, international normalized ratio (INR) 1.2). The rest of the laboratory results were unremarkable.

**Figure 1:**
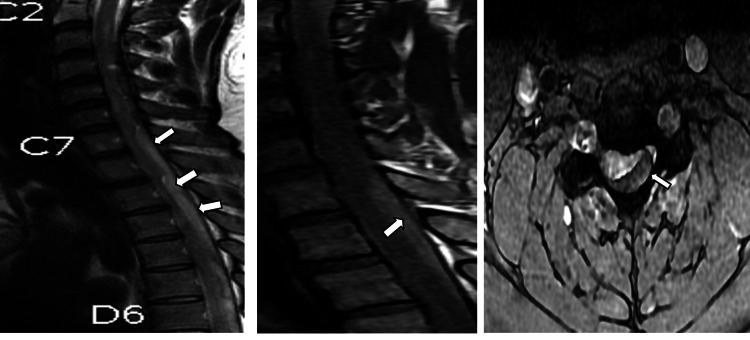
Long-segment cervicodorsal epidural hemorrhage A: Sagittal T2 image revealing a long-segment thick sheath of hyperintensity in the posterior epidural space extending from C2 to D7 level (arrows) B: Long-segment thick sheath of hyperintensity in the posterior epidural space extending from C2 to D7 level (arrows). The spinal cord was normal. C: Axial gradient echo (GRE) sequence showing posterior epidural hypointensity at the C4-C5 level which is mildly compressing and displacing the cord laterally to the right (arrows). Findings are suggestive of long-segment epidural hemorrhage.

**Figure 2:**
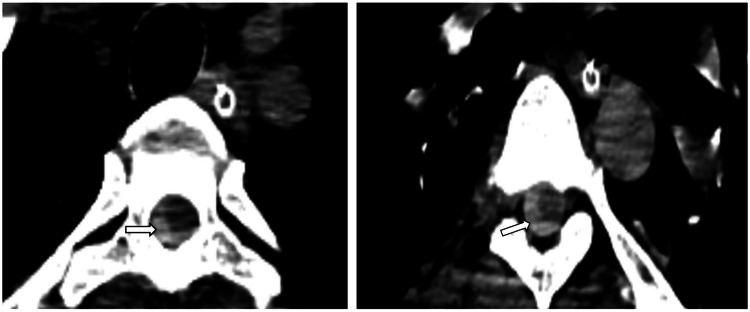
Axial non-contrast CT image showing epidural hemorrhage in the dorsal spine at D5-D6 and D6-D7 levels (arrows).

**Figure 3:**
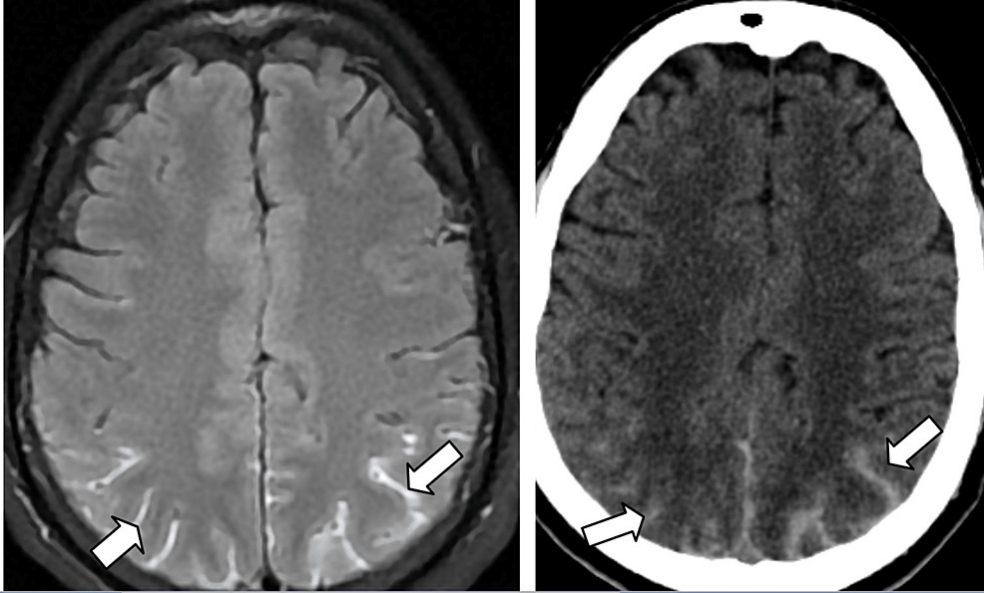
The MRI and CT images demonstrating subarachnoid hemorrhage in bilateral high parietal lobes (arrows) and also in occipital lobes with subtle dependent hemorrhage in bilateral occipital horns of the lateral ventricle (not shown here). A: Axial fluid-attenuated inversion recovery image showing sulcal hyperintensities in the bilateral high parietal lobes (arrows) B: Plain CT image showing mild subarachnoid hemorrhage in the bilateral high parietal lobes in the same patient (arrows)

The patient was started on intravenous antibiotics (doxycycline 100mg), analgesics, and other supportive medications. His weakness gradually progressed with the inability to walk or stand. The patient was referred for a spine MRI, which showed long-segment spinal epidural hemorrhage in the cervical and dorsal spine (up to D8 level) causing mild to moderate lateral compression and anterior displacement of the cord at these levels (Figures 1, 2). An MRI of the brain was also performed which revealed mild diffuse subarachnoid hemorrhage with subtle dependent hemorrhage in the bilateral occipital horns of the lateral ventricles which correlated with CT (Figure 3). After the radiology report, a neurosurgeon was consulted regarding the MRI findings and explained the grave risk and poor prognosis in relation to the radiological findings. Since the epidural hemorrhage was a long-segment (cervicodorsal spine epidural hematoma from C2 to D7 level) and not focal, surgical treatment was not possible and conservative management was advised. The patient was intubated due to unresponsiveness on day 10 of the dengue fever diagnosis. On the same day, the patient's family members decided to have the patient discharged against medical advice. Subsequently, the patient died of the disease.

## Discussion

Spinal epidural hemorrhage can be a rare complication of dengue virus infection and it can be focal and rarely with a long-segment involvement. However, in our case, the patient had compression of the cord and manifested as gradual or acute onset quadriparesis.

The cause of bleeding in dengue is multifactorial such as thrombocytopenia, platelet dysfunction [3], hepatic dysfunction, coagulopathy, and vasculopathy. In our case, moderate thrombocytopenia with platelet dysfunction could be a plausible mechanism for the longitudinal spinal epidural hemorrhage as the coagulation profile and liver function tests were unremarkable. In the literature, the thoracic spine is the most commonly involved site in epidural hemorrhage, and in our case, there was long-segment cervicodorsal spinal epidural hemorrhage and mild bilateral partial-occipital subarachnoid hemorrhage.

Few cases of spinal epidural hemorrhage as a complication of dengue fever have been reported in the literature. Suri et al. recently reported a case of dengue-induced cervical epidural hematoma during pregnancy [4]. Singh et al. reported a similar case where there was cord expansion and signal changes in the entire cord in addition to the D9 to D11 epidural hematoma [5]. In another case report of dengue, the extradural hematoma was found in the lumbar spine at levels L4 to L5 [6]. In another case, Fong et al. reported extensive longitudinal transverse myelitis with cervical epidural hematoma following a dengue virus infection [7].

Other radiological manifestations of dengue include gallbladder wall edema [8], third space loss (such as pleural effusion and ascites) and hepatosplenomegaly, meningoencephalitis in severe dengue fever [9]. Aseptic meningitis Guillain-Barre syndrome mononeuropathies/polyneuropathies, and myelitis are other atypical manifestations of dengue fever [10].

## Conclusions

Long-segment epidural hematoma in the spine is a rare complication of dengue virus disease and a high index of suspicion should be exercised in a dengue case with symptoms of upper or lower limb weakness. Hence, an early radiological diagnosis could help guide suitable patient management and prevent complications.
